# Activation of the Mevalonate Pathway in Response to Anti-cancer Treatments Drives Glioblastoma Recurrences Through Activation of *Rac-1*

**DOI:** 10.1158/2767-9764.CRC-24-0049

**Published:** 2024-06-25

**Authors:** Ling He, Angeliki Ioannidis, Carter J. Hoffman, Evelyn Arambula, Purva Joshi, Julian Whitelegge, Linda M. Liau, Harley I. Kornblum, Frank Pajonk

**Affiliations:** 1 Department of Radiation Oncology, David Geffen School of Medicine at UCLA, Los Angeles, California.; 2 Jonsson Comprehensive Cancer Center at UCLA, Los Angeles, California.; 3 Department of Psychiatry and Human Behavior, David Geffen School of Medicine at UCLA, Los Angeles, California.; 4 Department of Neurosurgery, David Geffen School of Medicine at UCLA, Los Angeles, California.

## Abstract

Glioblastoma (GBM) is the deadliest adult brain cancer. Under the current standard of care, almost all patients succumb to the disease and novel treatments are urgently needed. Recognizing that GBMs are addicted to cholesterol, past clinical trials have repurposed statins against GBM but failed. The purpose of this study was to test whether treatments that upregulate the cholesterol biosynthesis pathway in GBM would generate a metabolic vulnerability that can be exploited using statins and to determine the underlying mechanisms.

Effects of radiotherapy and temozolomide or dopamine receptor antagonists on the mevalonate pathway in GBM were assessed *in vitro* and *in vivo*. The impact of statins on self-renewal of glioma stem cells and median survival was studied. Branches of the mevalonate pathway were probed to identify relevant effector proteins.

Cells surviving combination treatments that converge in activating the immediate early response, universally upregulated the mevalonate pathway and increased stemness of GBM cells through activation of the Rho-GTPase *Rac-1*. Activation of the mevalonate pathway and *Rac-1* was inhibited by statins, which led to improved survival in mouse models of glioblastoma when combined with radiation and drugs that target the glioma stem cell pool and plasticity of glioma cells.

We conclude that a combination of dopamine receptor antagonists and statins could potentially improve radiotherapy outcome and warrants further investigation.

**Significance:**

Combination therapies that activate the mevalonate pathway in GBM cells after sublethal treatment enhance self-renewal and migratory capacity through *Rac-1* activation, which creates a metabolic vulnerability that can be further potentially exploited using statins.

## Introduction

Glioblastoma (GBM) is the deadliest adult brain cancer. The standard-of-care surgery followed by radiotherapy and temozolomide (TMZ) almost always fails and the tumors progress or recur, thus leading to unacceptably low 5-year survival rates. Despite iterations in radiotherapy protocols and techniques, introduction of targeted therapies and biologics, the survival rates have not changed in almost two decades and strategies against GBM have hit a critical barrier. Understanding mechanisms underlying this treatment resistance holds the key to developing novel approaches against GBM.

GBMs are thought to be governed by a hierarchical organization of tumor cells with a small number of glioma stem cells (GSC) at the top of this hierarchy, giving rise to the bulk of more differentiated glioma cells. GSCs have been reported to resist many chemotherapeutic agents (1) and are relatively resistant to ionizing radiation (2). Consequently, a portion of the GSC population survives treatment and can repopulate the tumor. This led to efforts specifically targeting the GSC population of GBMs but so far none of these attempts have successfully translated into the clinic.

Recently, we reported an additional novel aspect of GBM biology in response to radiation. Non-stem glioma cells surviving irradiation converted into glioma-initiating cells in a radiation dose-dependent manner (3). Dopamine receptor antagonists counteracting this phenomenon not only caused reductions in self-renewal capacity, clonogenicity, and cell viability in the GBM cells *in vitro*, but also exhibited antitumor effects on radioresistant cells as indicated by reduced self-renewal in secondary and tertiary glioma spheres (4, 5). Moreover, the combination of these drugs with radiation significantly improved median survival in patient-derived orthotopic xenograft (PDOX) and syngeneic mouse models of GBM (3–5). Yet, the efficacy was not 100% and bulk RNA sequencing revealed that surviving cells upregulated gene expression of the mevalonate pathway, followed by upregulation of its enzymatic function *in vitro* (4). This hinted at a novel metabolic vulnerability that could be further exploited by the addition of a statin, but it was unclear whether the same effects could be observed in the presence of tumor microenvironment *in vivo* and how the mevalonate pathway affects stemness of glioma cells. In the current study, we report that combination treatments that synergize with radiation in activating the immediate early response through the MAPK cascade upregulate the mevalonate pathway *in vivo*, thereby triggering repopulation of gliomas through prenylation of the Rho GTPase *Rac-1*.

## Materials and Methods

### Cell Lines

Primary human glioma cell lines were established and characterized at UCLA (Los Angeles, CA; ref. 6). Cells were propagated as gliomaspheres in serum-free conditions in ultra-low adhesion plates in DMEM/F12, supplemented with B27, EGF, bFGF, and heparin as described in ref. 7. The GL261 murine glioma cell line was a kind gift of Dr. William H. McBride (Department of Radiation Oncology at UCLA). GL261 cells were cultured in log-growth phase in DMEM supplemented with 10% FBS, penicillin and streptomycin (P/S). All cells were grown in a humidified atmosphere at 37°C with 5% CO_2_. The unique identity of all lines was confirmed by DNA fingerprinting (Laragen). All lines were routinely tested for *Mycoplasma* infection (#G238, Applied Biological Materials). For our experiments, we used cell lines that were at passages under 20.

### Animals

Six to eight weeks old C57BL/6 mice, or NOD-*scid* IL2Rgamma^null^ (NSG) originally obtained from The Jackson Laboratories were rederived, bred and maintained in a pathogen-free environment in the Division of Laboratory Animal Medicine, University of California, Los Angeles. A total of 2 × 10^5^ GL261-GFP-Luc or 3 × 10^5^ HK374-GFP-Luc cells were implanted into the right striatum of the brains of mice as described previously (3). Weight of the animals was recorded daily. Tumors were grown for 3 days for HK374 cells and 7 days for GL261 cells with successful grafting confirmed by bioluminescence imaging. Mice that developed neurologic deficits or lost 20% of their body weights requiring euthanasia were sacrificed.

### Ethics Statement

All animal experiments were approved by UCLA's Institutional Animal Care and Use Committee in accordance with all local and national guidelines for the care of animals.

### Drug Treatment

Mice implanted with HK374 cells were injected subcutaneously with quetiapine (QTP, 30 mg/kg) and/or intraperitoneally with simvastatin at 7 mg/kg on a 5-day on/2-day off schedule until they reached euthanasia endpoints. QTP was dissolved in acidified PBS (0.4% glacial acetic acid; 5 mg/mL). Simvastatin was dissolved in corn oil (2.5% DMSO, 5 mg/mL). Mice bearing GL261 tumors were injected intraperitoneally on a weekly basis either with ONC201 (50 mg/kg) and/or with atorvastatin (30 mg/kg) until they reached the euthanasia endpoint. ONC201 (Oncoceutics, Inc.) was dissolved in sterile saline (5.5 mg/mL). Atorvastatin was dissolved in corn oil (2.5% DMSO, 5 mg/mL).

To determine the optimal timing of the drug treatments during fractionated radiotherapy. Mice bearing GL261 tumors received five daily fractions of 3 Gy. In parallel, mice were treated with daily doses of either saline, QTP or QTP + atorvastatin. After completion of the radiation treatment, all animals were treated with QTP + atorvastatin until they reached criteria for euthanasia.

To explore the activation of MAPK pathway *in vitro*, HK374 cells were serum starved and the following day treated with a single dose of 10 Gy in the absence or presence of QTP (10 µmol/L), TMZ (1 mmol/L), or vincristine (250 nmol/L). Two hours after the irradiation, cell lysates were harvested for Western blotting.

To determine, which part of the mevalonate pathway synergizes with radiation and QTP to affect self-renewal, HK374 cells were plated under sphere-forming conditions in an *in vitro* limiting dilution assay. Cells were irradiated with a single dose of 4 Gy in the presence of QTP (10 µmol/L) and treated with inhibitors at indicated concentrations. All drugs were dissolved in DMSO at 10 mmol/L for stock and were replenished daily for 5 days.

### Migration Assay

HK374 monolayer cells were treated with 10 µmol/L QTP, or QTP and 1 µmol/L atorvastatin and irradiated with 0 or 4 Gy. A total of 48 hours after irradiation, cells were serum-starved for 12 hours. A total of 5 × 10^5^ cells were then plated into Transwell plates. After 24 hours, membranes were washed with PBS, cells fixed with 10% formalin, stained with 1% crystal violet, and counted using ImageJ.

### *In Vitro* Limiting Dilution Assay

For the assessment of self-renewal capacity *in vitro*, HK374 spheres were dissociated into single-cell suspension and seeded at clonal densities under serum-free conditions into non–tissue culture–treated 96-well plates in DMEM/F12 media, supplemented with SM1 Neuronal Supplement, EGF, bFGF and heparin. The next day, the cells were treated with drugs and 1 hour later irradiated with a single dose of 4 Gy. Growth factors were supplemented every 2 days. Glioma spheres were counted 10 days later and presented as the percentage of the initial number of cells plated.

### Irradiation

Cells and animals were irradiated using an experimental X-ray irradiator. For *in vivo* irradiation experiments, mice were anesthetized prior to irradiation with an intraperitoneal injection of 30 µL of a ketamine (100 mg/mL) and xylazine (20 mg/mL) mixture (4:1) and placed on their sides into an irradiation jig that allows for irradiation of the midbrain while shielding the esophagus, eyes, and the rest of the body. Details are described in Supplementary Materials and Methods.

### qRT-PCR

Total RNA cDNA synthesis and qRT-PCR were carried out using standard protocols (Supplementary Materials and Methods; primer sequences Supplementary Table S1).

### Cholesterol and Free Fatty Acid Quantification

A total of 3 × 10^5^ HK374-GFP-Luc sphere cells were intracranially implanted and grafted for 2 weeks to achieve the appropriate size of tumor to start with. Mice were irradiated with a single dose of 4 Gy and treated daily with either saline, QTP (30 mg/kg), TMZ (50 mg/kg), ONC201 (50 mg/kg), QTP + atorvastatin (30 mg/kg) or QTP + simvastatin (7 mg/kg). After 2 and 5 days of drug treatments, the mice were euthanized, and brain tumor samples were collected and weighed. PBS (20 µL/mg) was added to mince the brain tumor tissues with a pellet pestle tissue grinder (#749521-1590, DWK Life Sciences). Equal volumes of homogenized tumor specimens were subsequently subjected to Cholesterol/Cholesterol Ester-GLo assay and free fatty acid assay following the manufacturers’ instructions.

### Western Blotting

HK374 and HK217 monolayer cells were serum starved overnight and the following day treated with 10 µmol/L QTP, 1 mmol/L TMZ, or 250 nmol/L vincristine for 1 hour and then irradiated with a single dose of 10 Gy. Two hours after irradiation, the cells were lysed and proteins subjected to Western blotting following standard protocols (Supplementary Materials and Methods).

### Rac1 Pulldown Assay

HK374 monolayer cells were serum starved overnight, treated with QTP (10 µmol/L) or QTP + atorvastatin (1 µmol/L) and irradiated with a single dose of 4 Gy 1 hour after the drug treatment. A total of 48 hours after the treatment, the cells were lysed in 250 µL cell lysis buffer supplemented with protease inhibitors and subjected to a pulldown assay for activated Rac1 (#BK035, Cytoskeleton). Briefly, the protein concentration in each sample was determined using the bicinchoninic acid protein assay and 500 µg cell lysate was incubated with PAK-PBD beads at 4°C on a rotator for 1 hour. The beads were pelleted by centrifugation, washed with 500 µL wash buffer for two times, resuspended in 20 µL of 2x Laemmli sample buffer and boiled at 100°C for 5 minutes. The pulldown protein samples were then subjected to Western blotting with the whole cell lysate and His-tagged Rac1 protein (#RC01, Cytoskeleton) serving as the controls.

### siRNA and Extreme Limiting Dilution Analysis

HK374 monolayer cells were seeded into 6-well tissue-culture plates and transfected with scramble siRNA or Rac1 siRNA by incubation with RNAiMAX-siRNA Lipofectamine duplex in Opti-MEM medium overnight at 37°C with 5% CO_2_. The next day, cell culture medium was changed back to regular DMEM + 10% FBS + 1% P/S. Two days after the transfection, the cells were trypsinized and plated into the non–tissue culture–treated 96-well plates in DMEM/F12 media, supplemented with SM1 Neuronal Supplement, EGF, bFGF, and heparin. The next day, the cells were treated with DMSO or QTP (10 µmol/L) or QTP + atorvastatin (1 µmol/L) and irradiated with a single dose of 4 Gy 1 hour after the drug treatment. Growth factors were supplemented every 2 days. Glioma spheres were counted 5 days later (day 7 after siRNA transfection) and presented as the percentage to the initial number of cells plated. The glioma stem cell frequency was calculated using the extreme limiting dilution analysis (ELDA) software (8). Both protein and RNA samples were collected at day 2 and day 7 after transfection. Knockdown efficiency was confirmed by RT-PCR and Western blotting.

### Clonogenic Assay

Two days after the siRNA transfection, HK374 monolayer cells were trypsinized and plated at a density of 100 cells per well in a 6-well plate. The next day, the cells were treated with DMSO or QTP (10 µmol/L) or QTP + atorvastatin (1 µmol/L) and irradiated with a single dose of 4 Gy 1 hour after the drug treatment. The colonies were fixed and stained with 0.1% crystal violet 5 days later (day 7 after siRNA transfection). Colonies containing at least 50 cells were counted in each group.

### Microtubule Stain

HK374 monolayer cells were plated into 35 mm Poly-D-Lysine–coated dishes and the following day treated with QTP (10 µmol/L) or QTP + atorvastatin (1 µmol/L) and irradiated with a single dose of 4 Gy 1 hour after the drug treatment. A total of 48 hours later, cells were stained with ViaFluor 488 live cell microtubule stains following manufacturer's instructions. Specifically, the cells were washed with PBS and incubated with medium containing probes (0.5 × ViaFluor dye + 100 µmol/L verapamil) at 37°C for 30 minutes. Fluorescent images were then acquired using a confocal microscope (Nikon A1).

### Mass Spectrometry

Sample Preparation: Whole blood from mice was centrifuged to isolate plasma. Atorvastatin or simvastatin was isolated by liquid-liquid extraction from plasma: 50 µL plasma was added to 2 µL internal standard and 100 µL acetonitrile. Mouse brain tissue was washed with 2 mL cold saline and homogenized using a tissue homogenizer with fresh 2 mL cold saline. Atorvastatin or simvastatin was then isolated and reconstituted in a similar manner by liquid-liquid extraction: 100 µL brain homogenate was added to 2 µL internal standard and 200 µL acetonitrile. The samples were centrifuged, supernatant removed and evaporated by a rotary evaporator and reconstituted in 100 µL 50:50 water:acetonitrile.

Atorvastatin brain concentrations were adjusted by 1.4% of the mouse brain weight for the residual blood in the brain vasculature as described by Dai and colleagues (9). Details on instrumentation and setting are described in Supplementary Materials and Methods).

### Statistical Analysis

Unless stated otherwise all data shown are represented as mean ± SEM of at least three biologically independent experiments. A *P*-value of ≤0.05 in an unpaired two-sided *t* test, one-way or two-way ANOVA indicated a statistically significant difference. For Kaplan–Meier estimates, a *P*-value of 0.05 in a log-rank test indicated a statistically significant difference.

### Data Availability

All data and methods are included in the article or in the Supplementary Materials and Methods. Patient-derived cell lines will be made available upon reasonable request to the corresponding author.

## Results

### Upregulation of the Mevalonate Pathway in Response to Anti-cancer Treatments *In Vivo* is Restricted to Glioma Cells

We previously reported that combinations of radiation and dopamine receptor antagonists upregulated genes in the mevalonate pathway and increased cholesterol biosynthesis in GBM cells *in vitro* (3, 4). Genes in this pathway are regulated by the sterol regulatory element-binding protein 2 (*SREBP-2* also called *SREBF-2*), a transcription factor under control of the MAPK cascade through *AP-1*, a pathway well known to be activated during the immediate-early response to radiation (10). Its components *JunB*, *JunD*, and *FosB*, are all part of the first-order regulatory network of *SREBP-2*. *JunB* and *JunD* have weak transactivation domains and can be considered repressors in the presence of the strong transcriptional activator *FosB*. All three are regulated by *GSK-3* (11), the kinase downstream of dopamine receptors, thus explaining synergistic effects of radiation and dopamine receptor antagonists on the mevalonate pathway.

**FIGURE 1:**
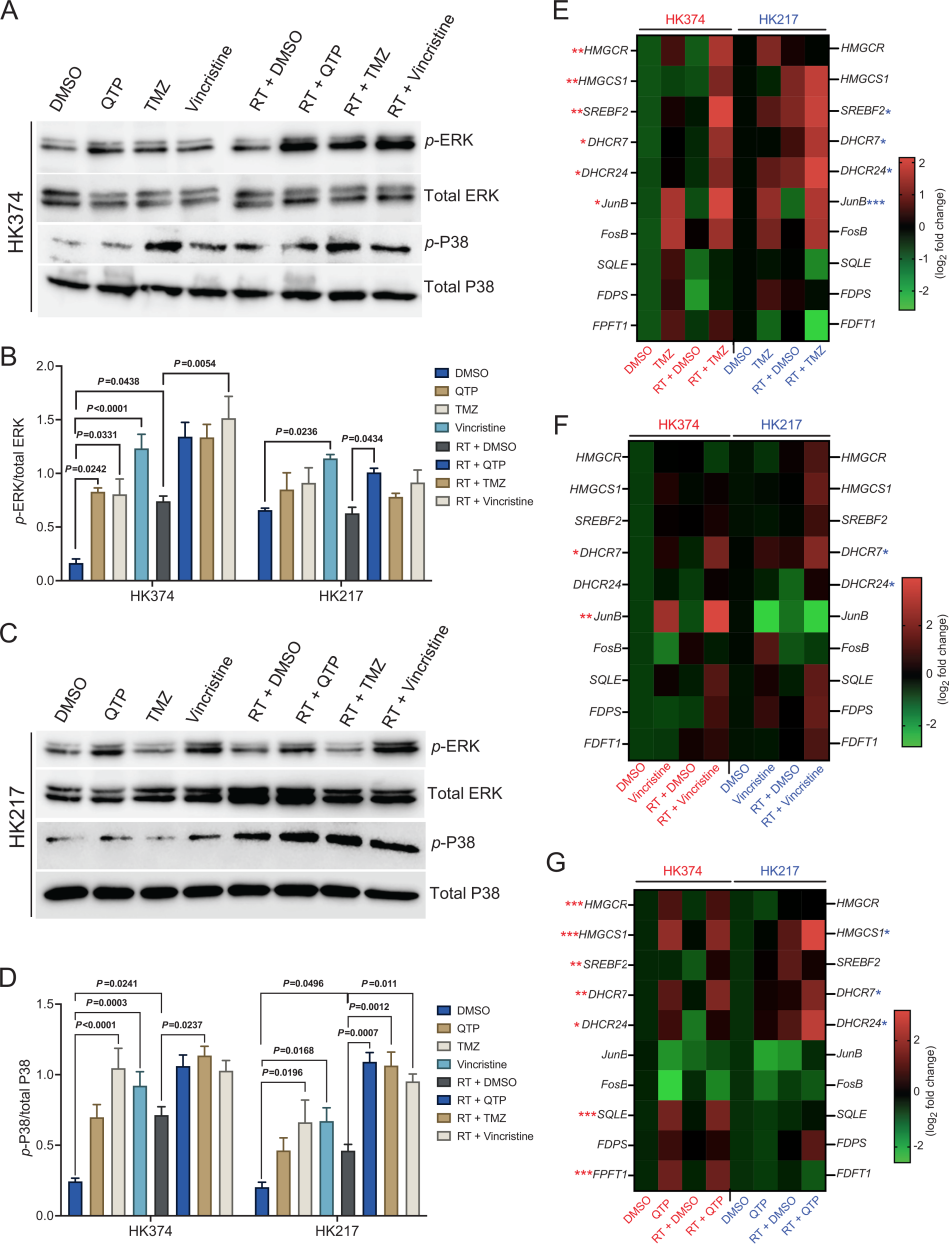
Combination therapies that converge on the immediate-early response to radiation through the MAPK cascade universally upregulate the mevalonate pathway. **A** and **C,** Western blotting of *p*-ERK, total ERK, *p*-P38, and total P38 in patient-derived GBM HK374 and HK217 cell lines upon radiation (RT) in combination with QTP (10 µmol/L) or TMZ (1 mmol/L) or vincristine (250 nmol/L) at 2 hours after treatment. **B** and **D,** The densitometry measurements of *p*-ERK/total ERK and *p*-P38/total P38 using Image J. **E,** Heat map showing the results of qRT-PCR for the cholesterol biosynthesis–related genes in both HK374 and HK217 cells treated with radiation in the presence or absence of TMZ (1 mmol/L) for 2 consecutive days. **F,** Heat map showing the results of qRT-PCR for the cholesterol biosynthesis–related genes in both HK374 and HK217 cells treated with radiation in the presence or absence of vincristine (250 nmol/L) for 24 hours. **G,** Heat map showing the results of qRT-PCR for the cholesterol biosynthesis–related genes in both HK374 and HK217 cells treated with radiation in the presence or absence of QTP (10 µmol/L) for 2 consecutive days. All experiments have been performed with at least three biological independent repeats. *P-*values were calculated using one-way ANOVA. The *P*-values listed in the heat maps were from the comparison of RT + DMSO with RT + TMZ (E) or RT + Vincristine (F) or RT + QTP (G). *, *P* < 0.05; **, *P* < 0.01; ***, *P* < 0.001.

TMZ, part of the current standard of care against GBM is known to activate the MAPK cascade in GBM (12). Likewise, vincristine, a component of the Procarbazine, Lomustine, and Vincristine (PCV) chemotherapy regimen against GBM, activates this pathway (13). Western blots of patient-derived HK374 and HK217 glioma cells (Table 1) confirmed that radiation, QTP (a dopamine receptor antagonist), TMZ, and vincristine activated the MAPK cascade in our model system (Fig. 1A–D; Supplementary Fig. S1A–S1C). Next, we tested whether radiation in combination with TMZ, vincristine, or QTP would also upregulate the mevalonate pathway. As expected, combining radiation and TMZ upregulated gene expression of key enzymes in this pathway (Fig. 1E). Similar results were found when combining radiation and vincristine (Fig. 1F). We have previously reported the upregulation of mevalonate pathway in HK374 cells upon combination treatment of radiation plus QTP (4). Here, similar results were observed in HK217 cells (Fig. 1G). This indicated that combination therapies that converge on the immediate-early response to radiation (14) through activation of the MAPK cascade universally upregulate the mevalonate pathway, thereby creating a metabolic vulnerability in cells surviving the sublethal DNA damage caused by radiation.

**TABLE 1:** Patient demographics and TCGA-classification of GBM subtypes

Line	Origin	Age	Sex	TCGA subtype	Culture P53 CN	EGFRvIII	PTEN	MGMT
HK-374	Primary GBM	45	M	classical	Loss “mosaic”	Positive	Positive	not methylated
HK-217	Primary GBM	81	M	proneural	wt	Negative	Negative	methylated

**FIGURE 2:**
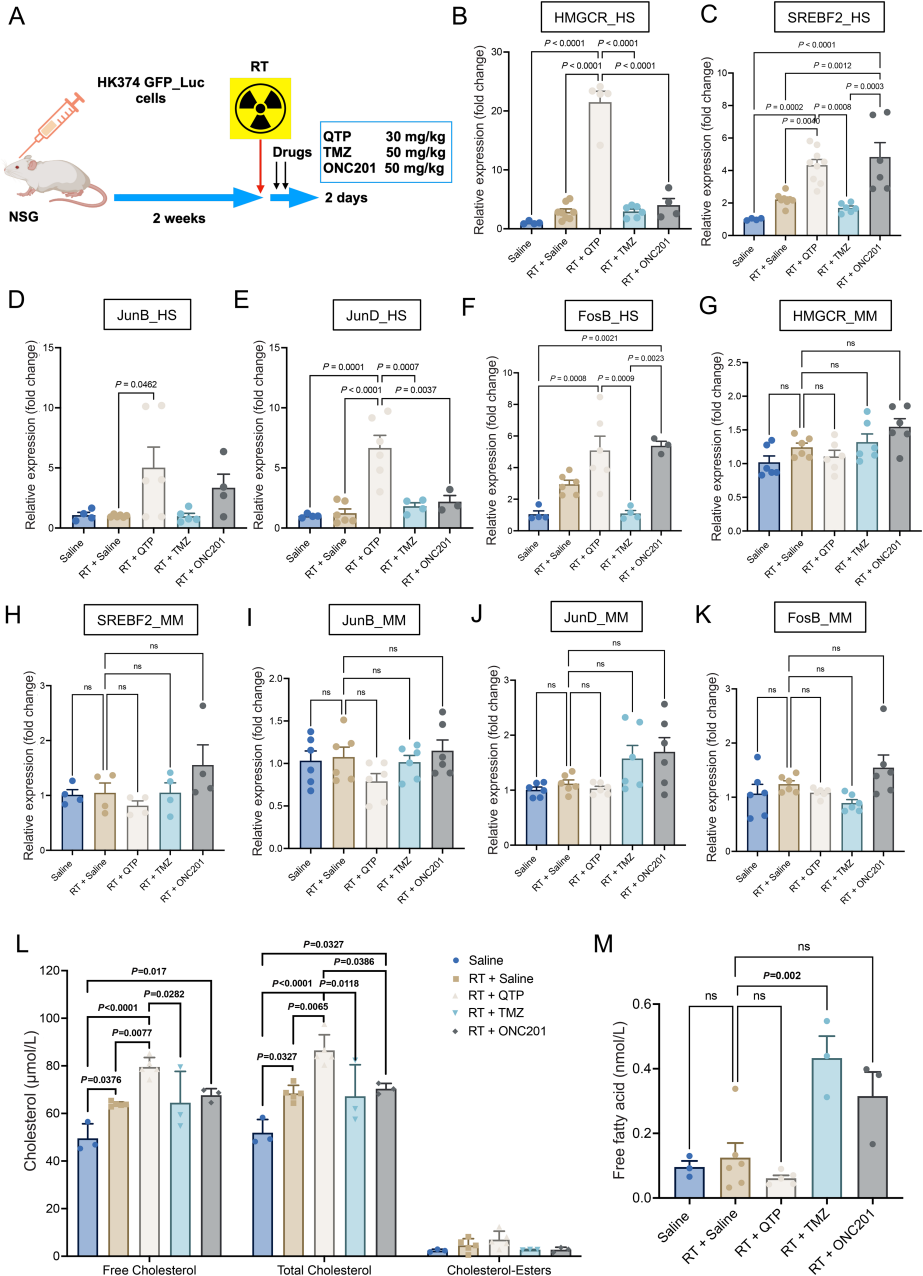
Upregulation of the mevalonate pathway in response to anti-cancer treatments *in vivo* is restricted to glioma cells. **A,** Schematic of the experimental design underlying Fig. 2. **B–F,** qRT-PCR for the cholesterol biosynthesis–related genes in the tumor specimen harvested from the PDOX GBM mouse model at day 2 after the treatment of radiation in combination with QTP (30 mg/kg) or TMZ (50 mg/kg) or ONC201 (50 mg/kg) or solvent control using the human-specific primers. **G–K,** qRT-PCR for the cholesterol biosynthesis–related genes in the tumor specimen harvested from the PDOX GBM mouse model at day 2 after the treatments using the mouse-specific primers. **L,** The levels of total, free cholesterol and cholesterol-esters in the tumors harvested from the PDOX GBM mouse model at day 2 after the treatments. **M,** The concentration of free fatty acid in the tumors harvested from the PDOX GBM mouse model at day 2 after the treatments. All experiments have been performed with at least three biological independent repeats. *P-*values were calculated using one-way ANOVA. ns: not significant.

Our previous studies investigated the effects of radiation and dopamine receptor antagonists on the mevalonate pathway in patient-derived GBM lines *in vitro* (4). To test whether this effect could also be observed in the presence of a tumor microenvironment, we next implanted HK374 glioma cells into the striatum of NSG mice. After 2 weeks of grafting and tumor growth, mice were irradiated and treated with two consecutive doses of QTP, TMZ, or ONC201 (Fig. 2A). Tumor tissue was harvested, mRNA extracted and subjected to qRT-PCR using human- and mouse-specific primers for genes in the mevalonate pathway. Gene expression of enzymes in the mevalonate pathway in tumor cells confirmed data obtained in our *in vitro* studies showing upregulation of gene expression in response to radiation in combination with QTP. However, upregulation of these metabolic enzyme genes was not observed when radiation was combined with TMZ, while combination of radiation with ONC201 showed some effects, with increased expression of SREBF2 and *FosB* (Fig. 2B–F). Using mouse-specific primers for the corresponding murine genes we did not observe an upregulation of these genes in normal cells in the tumor microenvironment, thus indicating that this response was specific to GBM cells (Fig. 2G–K).

In *in vitro* shotgun lipidomics experiments, we previously demonstrated that radiation in combination with QTP upregulated cholesterol and free fatty acid production (4). To test whether these pathways were also functionally upregulated in response to the combination therapy *in vivo*, we next quantified cholesterol and free fatty acid levels in tumor tissues. Compared with solvent control, radiation alone significantly augmented free and total cholesterol levels, and the additional QTP treatment further upregulated those cholesterol levels in the tumor tissue, but not with the treatments of TMZ nor ONC201 (Fig. 2L). However, free fatty acid level was not significantly affected by combination therapy of radiation with QTP in this *in vivo* setting (Fig. 2M), suggesting that the mevalonate pathway was the one not only transcriptionally but also functionally activated. However, the increases in cholesterol synthesis were unexpectedly small, thus hinting that other branches of the mevalonate pathway could be involved in mediating the survival of GBM cells.

### Statins Reduce Treatment-induced Upregulation of Cholesterol Biosynthesis in PDOXs

**FIGURE 3:**
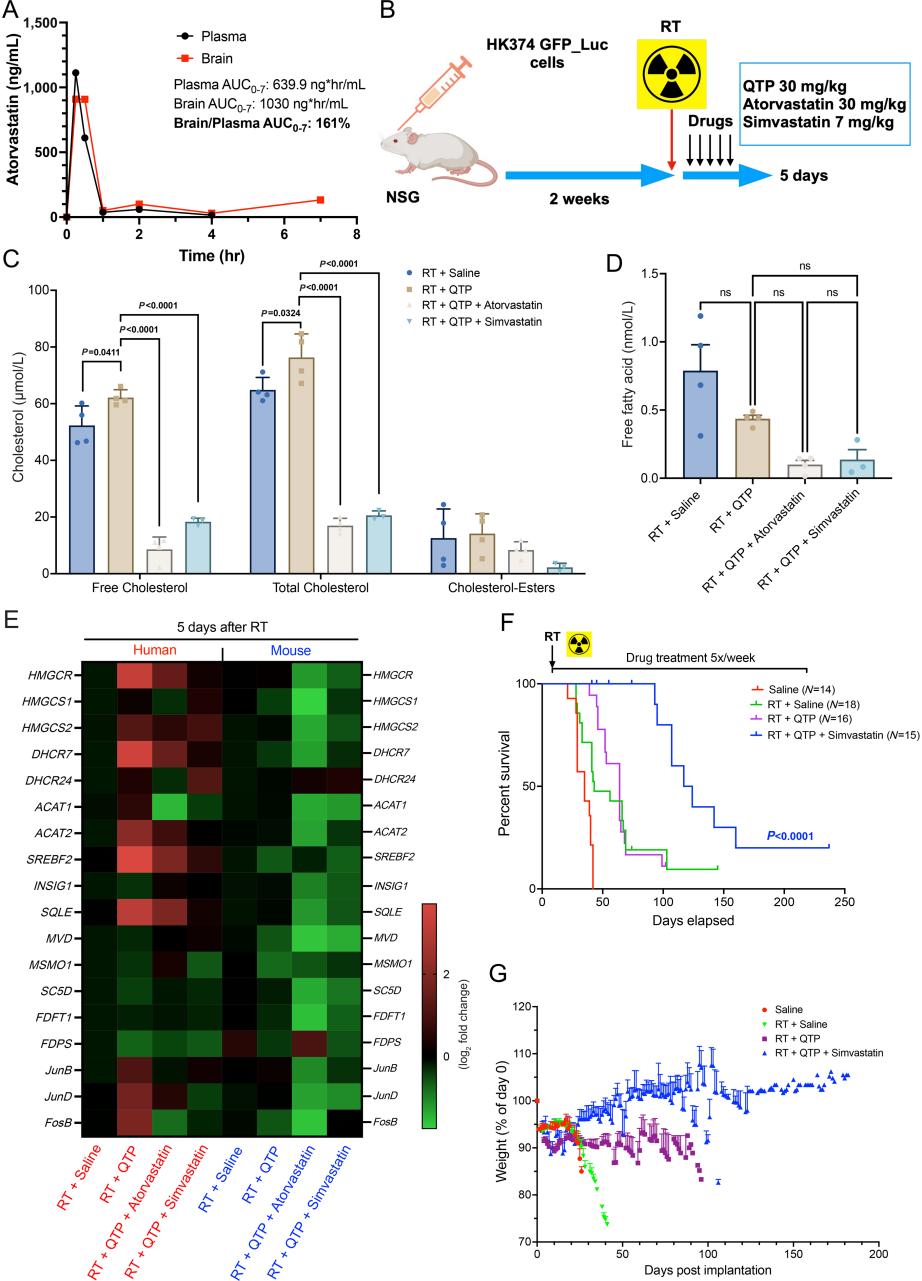
Statins reduce treatment-induced upregulation of cholesterol biosynthesis in PDOXs. **A,** Brain and plasma levels of atorvastatin in C57BL/6 mice after a single injection (atorvastatin – 30 mg/kg, i.p.). **B,** Schematic of the experimental design underlying Fig. 3. **C,** The levels of total, free cholesterol and cholesterol-esters in the tumors harvested from the PDOX GBM mouse model at day 5 after the treatment of radiation in combination with QTP (30 mg/kg), atorvastatin (30 mg/kg) or simvastatin (7 mg/kg). **D,** The concentration of free fatty acid in the tumors harvested from the PDOX GBM mouse model at day 5 after the treatments. **E,** Heat map showing the results of qRT-PCR for the cholesterol biosynthesis–related genes in the tumor specimen harvested from the PDOX GBM mouse model at day 5 after the treatments using the human- and mouse-specific primers. **F,** Survival curves for NSG mice implanted intracranially with 3 × 10^5^ HK374-GFP-Luciferase glioma cells and grafted for 3 days. Mice were irradiated and treated with saline or QTP (30 mg/kg, s.c., 5-day on/2-day off schedule) or triple combination of radiation plus QTP and simvastatin (7 mg/kg, i.p., 5-day on/2-day off schedule) continuously until they reached the study endpoint. log-rank (Mantel–Cox) test for comparison of Kaplan–Meier survival curves. **G,** Weight curves for the NSG mice in different treatment groups. All experiments have been performed with at least three biological independent repeats. *P-*values were calculated using one-way ANOVA. ns: not significant.

Statins are a class of FDA-approved drugs that target 3-hydroxy-3-methylglutaryl-CoA reductase (*HMGCR*), the rate-limiting enzyme in the mevalonate pathway. Designed to lower cholesterol levels in the periphery, data on their blood–brain barrier (BBB) penetration in the literature are sparse and often conflicting. Our *in vivo* data indicated that the addition of statins to a combination of radiation and QTP could significantly improve median survival in mouse models of GBM (4). We had previously used atorvastatin at a dose of 30 mg/kg in mice, and at this dose we were able to detect atorvastatin in brain tissue and plasma of mice with a brain to plasma ratio of 1.6 (Fig. 3A), which suggested that atorvastatin penetrates the BBB. We then employed a machine learning–based computational quantitative model (15) to predict the BBB permeability of all FDA-approved statins as well as their involvements in clinical trials against brain tumors (Table 2). We noticed that a superior BBB penetration was predicated for another statin—simvastatin—with a logBB of −0.21 versus −1.35 for atorvastatin. However, simvastatin is a prodrug and subject to a first-pass effect and rapid conversion into its active form, simvastatin hydroxy acid, which is predicted to cross the BBB (logBB −0.67). To test whether the addition of statins would alter the mevalonate pathways in PDOXs, we grafted the HK374 cells and let the tumor graft and grow for 2 weeks, mice were then treated with radiation and five consecutive doses of QTP in the presence or absence of atorvastatin or simvastatin (Fig. 3B). The results showed that both—atorvastatin and simvastatin—reduced free and total cholesterol (Fig. 3C) but not free fatty acid levels (Fig. 3D) in PDOXs. This indicated that both statins crossed the BBB in their active forms in pharmacologically relevant concentrations or that there is sufficient diminution of the BBB in xenografts to let statins reach the tumor tissue. Transcriptionally, the addition of statins decreased mevalonate pathway gene expression in both tumor and normal tissues, when compared with combination treatment of radiation and QTP (Fig. 3E).

**TABLE 2:** Prediction of BBB permeability of statins and their involvements in brain tumor clinical trials

Drug name	SMILES	2D depiction	LogBB value[15]	Clinical trials	Tumor type	Interventions
Simvastatin (Zocor®)	CCC(C)(C)C(=O)O[C@H]1C [C@@H](C)C=C2C=C[C@H] (C)[C@H](CC[C@@H]3C [C@@H](O)CC(=O)O3)[C @H]21	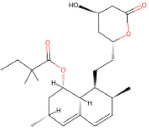	−0.20879	NCT01764451	Cerebral Cavernous Hemangioma	Simvastatin
				NCT02104193	Brain Metastases	Simvastatin + Radiation therapy
Atorvastatin (Lipitor)	CC(C)c1c(C(=O)Nc2ccccc2) c(-c2ccccc2)c(-c2ccc(F) cc2)n1CC[C@H](O)C [C@H](O)CC(=O)O	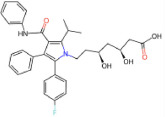	−1.34826	NCT02603328	Cerebral Cavernous Hemangioma	Atorvastatin
				NCT02029573	Glioblastoma Multiforme	Atorvastatin + Temozolomide + Radiotherapy
Rosuvastatin (Crestor)	CC(C)c1nc(N(C)S(C)(=O)=O) nc(-c2ccc(F)cc2)c1C=CC(O) C[C@@H](O)CC(=O)O	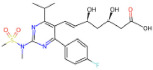	−1.14247	N		
Fluvastatin (Lescol)	CC(C)n1c(C=CC(O)C[C@H](O) CC(=O)O)c(-c2ccc(F) cc2)c2ccccc21	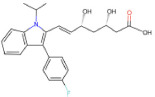	−1.04942	N		
Lovastatin (Mevacor)	CC[C@H](C)C(=O)O[C@H]1C [C@@H](C)C=C2C=C[C@H] (C)[C@H](CO[C@@H]3C[C@ @H](O)CC(=O)O3)[C@H]21	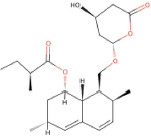	−0.30716	NCT04297033	Cerebral Arteriovenous Malformation	Lovastatin
Pitavastatin (Livalo)	O=C(O)C[C@H](O)CC(O)C=C c1c(C2CC2)nc2ccccc2c1-c1ccc(F)cc1	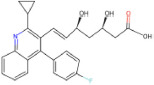	−1.12192	NCT05977738	Glioblastoma Multiforme; Recurrent Glioblastoma	Pitavastatin calcium
Pravastatin (Pravachol)	CC[C@H](C)C(=O)O[C@H]1C [C@H](O)C=C2C=C[C@H] (C)[C@H](CC[C@@H](O)C [C@@H](O)CC(=O)O)[C@H]21	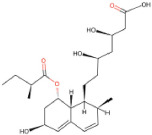	−0.91495	N		

Abbreviations: BBB, blood-brain barrier; SMILES, simplified molecular-input line-entry system; LogBB value, a logarithmic ratio of the concentration of a drug in the brain to its concentration in the blood.

Clinical trial data were assessed through clinicaltrials.gov at March 21, 2024.

### Statins Improve Median Survival in Mouse Models of GBM Undergoing Single dose or Fractionated Irradiation

**FIGURE 4:**
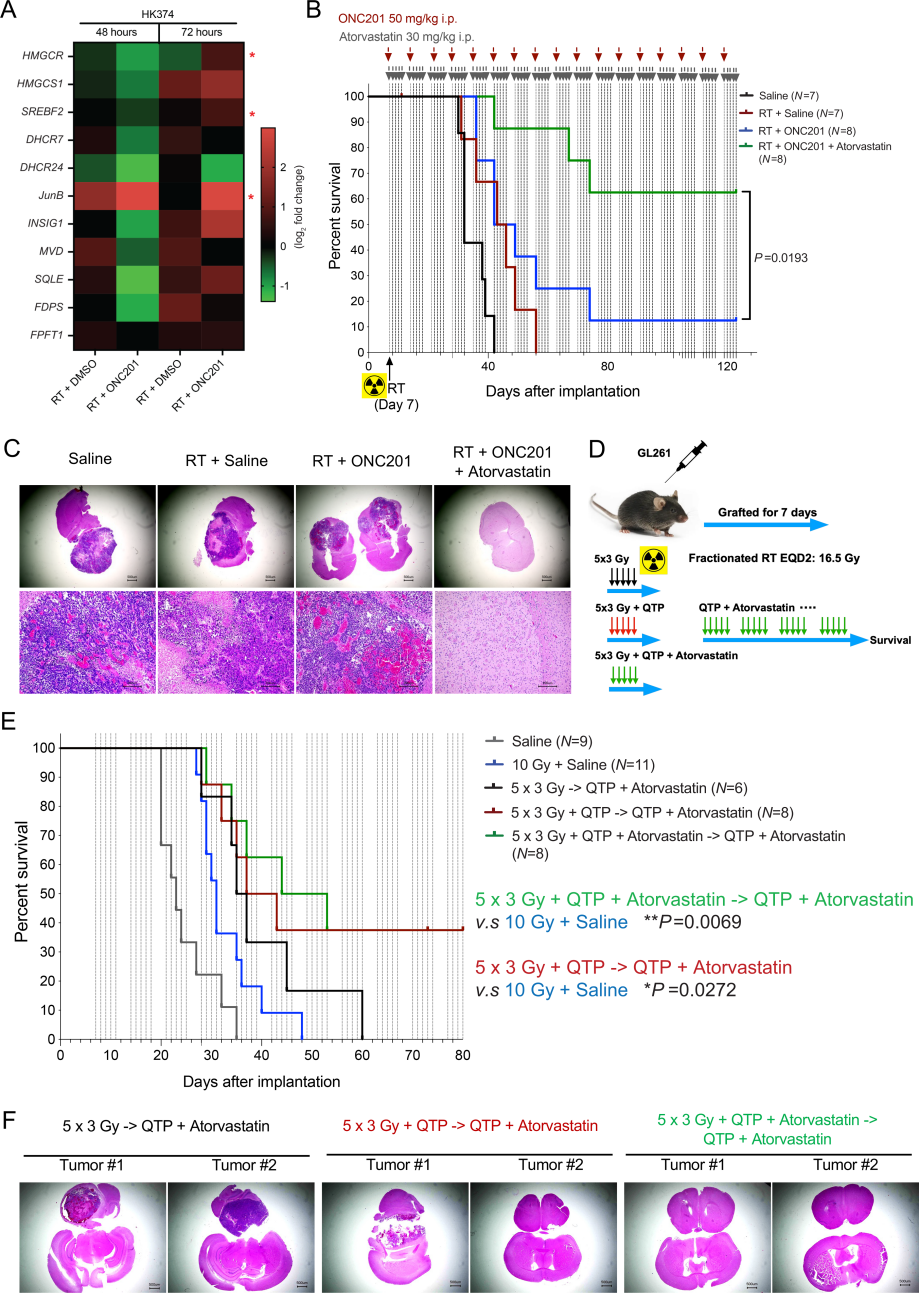
Statins improve median survival in mouse models of GBM undergoing fractionated irradiation. **A,** Heat map showing the results of qRT-PCR for the cholesterol biosynthesis–related genes in HK374 cells treated with radiation in the presence or absence of ONC201 (a single treatment of 2.5 µmol/L) at 48 and 72 hours. *P-*values were calculated using unpaired Student *t* test for A. *, *P* < 0.05. **B,** Survival curves for C57BL/6 mice implanted intracranially with 2 × 10^5^ GL261-GFP-Luciferase mouse glioma cells and grafted for 7 days. Mice were irradiated and weekly treated with saline or ONC201 (50 mg/kg, i.p.) or triple combination of radiation plus ONC201 (weekly) and atorvastatin (30 mg/kg, i.p., 5-day on/2-day off schedule) continuously until they reached the study endpoint. log-rank (Mantel–Cox) test for comparison of Kaplan–Meier survival curves. **C,** H&E-stained coronal sections of the C57BL/6 mice brains implanted with GL261-GFP-Luc cells which were irradiated and treated continuously with ONC201 in the presence or absence of atorvastatin until they met the criteria for study endpoint. **D,** Schematic of the experimental design of fractionated irradiation in syngeneic mouse model of GBM. **E,** Kaplan–Meier survival curves for C57BL/6 mice implanted intracranially with GL261-GFP-Luciferase mouse glioma cells and treated with either a single fraction of 0 or 10 Gy or five daily fractions of 3 Gy each and daily doses of either saline, QTP (30 mg/kg, s.c.), or QTP plus atorvastatin (30 mg/kg, i.p.). After completion of the radiation treatment all animals were treated with QTP plus atorvastatin until they reached criteria for euthanasia. log-rank (Mantel–Cox) test for comparison of Kaplan–Meier survival curves. **F,** H&E-stained coronal sections of the C57BL/6 mice brains from the groups of 5 × 3 Gy → QTP + atorvastatin, 5 × 3 Gy + QTP → QTP + atorvastatin and 5 × 3 Gy + QTP + atorvastatin → QTP + atorvastatin.

In previous *in vitro* studies, we reported that the dopamine receptor antagonists trifluoperazine (TFP) and QTP prevented radiation-induced phenotype conversion of non-stem glioma cells into glioma stem cells, but induced gene expression of the mevalonate pathway in surviving cells, thereby creating a metabolic vulnerability that could be exploited with the use of atorvastatin to further improve median survival (4). At 7 mg/kg, equivalent to the recommended human dose of 40 mg, simvastatin also significantly prolonged the median survival of glioma-bearing mice when combined with radiation and QTP (RT + QTP: 64 days vs. RT + QTP + simvastatin: 120.5 days, *P* < 0.0001, log-rank test; Fig. 3F) and was well tolerated with animals gaining weight during treatment (Fig. 3G). We previously reported that the atypical dopamine receptor 2/3 antagonist and caseinolytic protease P (ClpP) activator ONC201 (16), now in clinical trials against pediatric glioma, also prevented radiation-induced phenotype conversion of non-stem glioma cells into glioma stem cells and prolonged median survival in mouse models of GBM (5). Here we report that like TFP and QTP, ONC201 in combination with radiation also upregulates the mevalonate pathway, although with different kinetics, reflective of the longer half-life of ONC201 (Fig. 4A). Importantly, the addition of a statin significantly improved median survival compared with animals treated with radiation and ONC201 alone (RT + ONC201 + atorvastatin vs. RT + ONC201, *P* = 0.0193, log-rank test; Fig. 4B), and led to smaller tumors or no signs of residual tumor in some animals as shown in hematoxylin and eosin (H&E) staining (Fig. 4C).

Next, we repeated the experiment with fractionated irradiation (five daily fractions of 3 Gy) in GL261 glioma bearing mice. In parallel, mice were treated with daily doses of either saline, QTP, or QTP plus atorvastatin. After completion of the radiation treatment, all animals were treated with QTP plus atorvastatin until they reached criteria for euthanasia (Fig. 4D). Kaplan–Meier estimates for the three treatment arms did not differ significantly but showed a trend for improved median survival for animals receiving QTP or QTP + atorvastatin during radiation treatment (Fig. 4E) in line with QTP preventing radiation-induced phenotype conversion of non-stem GBM cells into GSCs and atorvastatin affecting the mevalonate pathway in surviving cells. Histologically, the triple combination of radiation, QTP and atorvastatin led to smaller tumor sizes or no signs of residual tumor in some animals (Fig. 4F).

### Treatment-induced Upregulation of the Mevalonate Pathway in Glioma Affects Stemness Through Prenylation Of *Rac-1*

**FIGURE 5:**
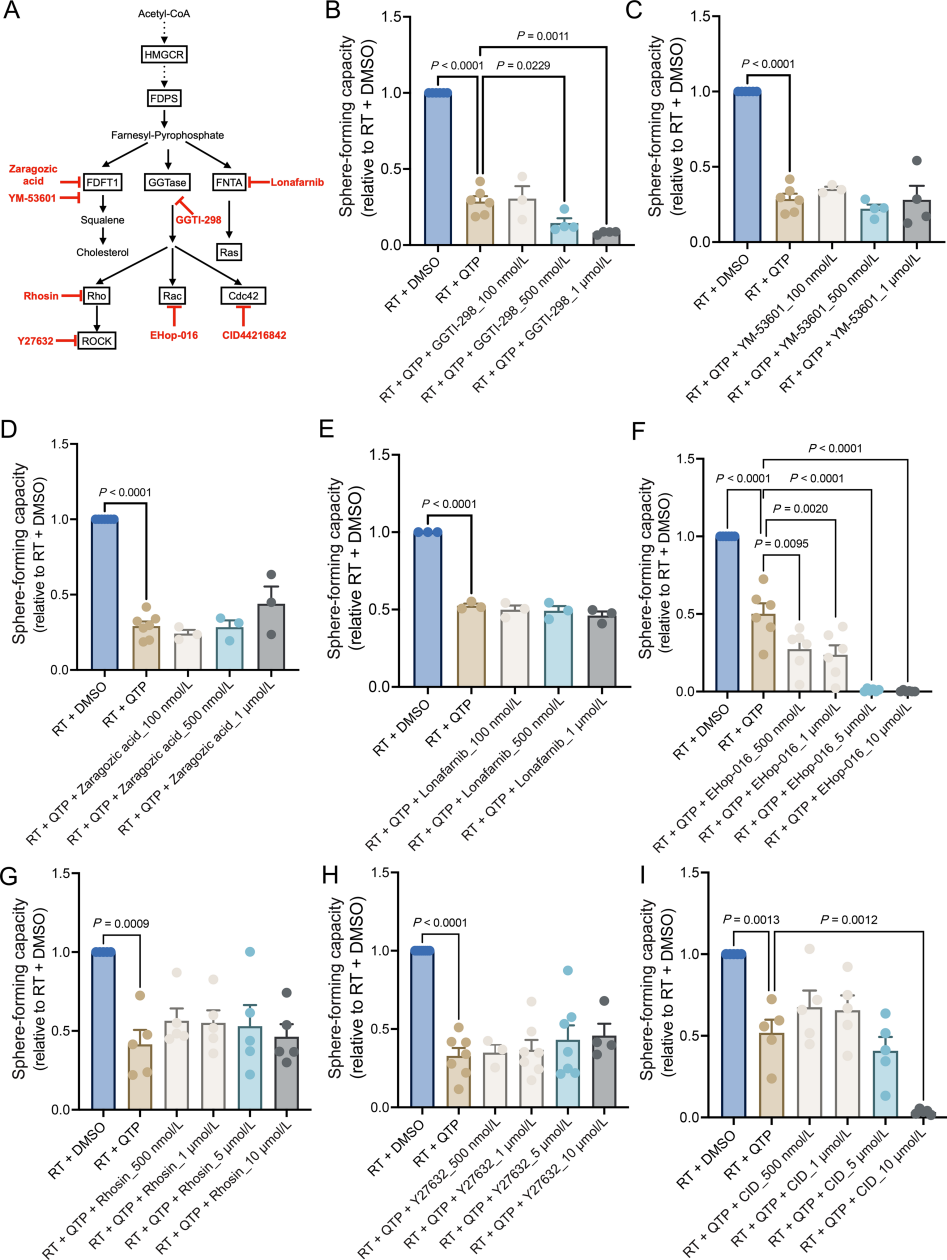
Identification of Rac1 as the potential contributor for maintaining the stemness of surviving glioma cells. **A,** Cholesterol biosynthesis pathway and the key selected inhibitors. **B–E,** Sphere-forming capacity of HK374 spheres treated either with GGTI-298 (GGTase inhibitor) or YM-53601 (squalene synthase inhibitor) or Zaragozic acid (squalene synthase inhibitor) or Lonafarnib (farnesyltransferase inhibitor) at 100, 500 nmol/L, 1 µmol/L concentrations when combined with radiation and QTP (10 µmol/L). **F–I,** Sphere-forming capacity of HK374 spheres treated either with Ehop-016 (Rac GTPase inhibitor) or Rhosin (RhoA-specific inhibitor) or Y27632 (ROCK1/2 inhibitor) or CID44216842 (Cdc42-selective inhibitor) at 500 nmol/L, 1, 5, 10 µmol/L concentrations when combined with radiation and QTP. All experiments have been performed with at least three biological independent repeats. *P-*values were calculated using one-way ANOVA.

The mevalonate pathway is the fundamental pathway in cholesterol biosynthesis. While the uptake of exogenous cholesterol has been shown to be essential for glioma (17), other metabolites in the mevalonate pathway contribute to normal cell function through farnesylation and geranylgeranylation to the prenylation of small GTPases (18, 19). To determine which of the pathway components’ upregulation contributes to glioma stemness, we employed an inhibitor-based approach (Fig. 5A). Using clonal sphere-forming capacity assay, we found that inhibition of geranylgeranylation (Fig. 5B) but not inhibition of the cholesterol biosynthesis (Fig. 5C and D) or farnesylation (Fig. 5E) further reduced sphere formation when combined with radiation and QTP.

Because geranylgeranylation of Rho GTPases is fundamental for their downstream effects on cytoskeletal reorganization (20), which mediates the interaction of cancer stem cells (CSC) with the tumor microenvironment, maintenance of stemness, and CSC migration (21) we next tested which Rho GTPase would affect the self-renewal capacity of GBM cells (Fig. 5A). Inhibition of Rac GTPases (Fig. 5F) but not Rho or Cdc42 GTPases (Fig. 5G–I) significantly reduced sphere formation in a dose-dependent fashion when combined with radiation and QTP.

**FIGURE 6:**
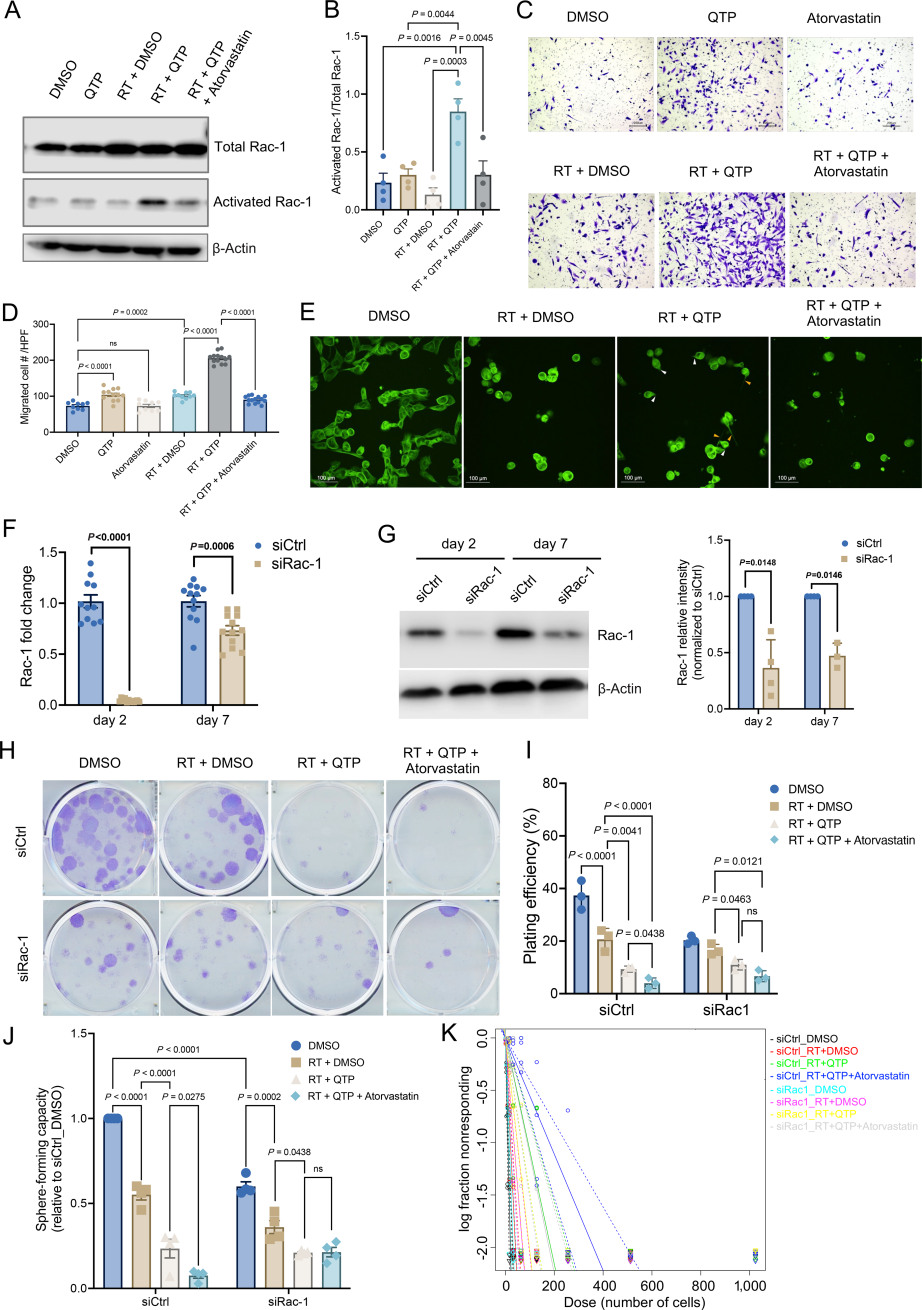
Treatment-induced upregulation of the mevalonate pathway in glioma affects stemness through prenylation of Rac1. **A,** HK374 cells were treated with radiation in the presence or absence of QTP (10 µmol/L) and/or atorvastatin (1 µmol/L) for 48 hours. The activated Rac1 was immunoprecipitated by 10 µg PAK-PBD agarose beads from the whole cell lysates and subjected to immunoblotting against Rac1, along with the total proteins. His-tagged Rac1 protein serves as the positive control. **B,** The densitometry measurements of activated Rac1/total Rac1 using Image J. **C,** Transwell migration assay of HK374 cells pretreated with radiation in the presence or absence of QTP (10 µmol/L) and/or atorvastatin (1 µmol/L) for 48 hours. **D,** The quantification of migrated cells using Image J. **E,** Confocal images of microtubules in HK374 cells treated with radiation in the presence or absence of QTP (10 µmol/L) and/or atorvastatin (1 µmol/L) for 48 hours. White arrowheads: filopodia. Yellow arrowheads: TNTs. **F** and **G,** Rac1 knockdown efficiency was evaluated at both mRNA (qRT-PCR) and protein (Western blotting) levels at day 2 and day 7 after siRNA transfection. β-actin was used as the loading control and the densitometry measurements of Rac1 were performed using Image J. **H** and **I,** Clonogenic assay of siCtrl or siRac1 transfected HK374 cells treated with radiation in the presence or absence of QTP (10 µmol/L) and/or atorvastatin (1 µmol/L) for 7 days and the resulting plating efficiencies. **J,** Sphere-forming capacity of siCtrl or siRac1 HK374 spheres treated with radiation in the presence or absence of QTP (10 µmol/L) and/or atorvastatin (1 µmol/L). **K,** log fraction plot generated by extreme limiting dilution assay (ELDA), where the *y* axis “log fraction nonresponding” indicates frequency of cells incapable of forming clonal spheres and the *x* axis “dose (number of cells)” indicates number of cells per mL. The slope of the line is the log-active cell fraction, and dotted lines give the 95% confidence interval. All experiments have been performed with at least three biological independent repeats. *P-*values were calculated using one-way ANOVA for B and D, unpaired Student *t* tests for F and G; two-way ANOVA for I and J. ns: not significant.

In agreement with our inhibitor studies, *Rac-1* pulldown assays revealed a significant activation of *Rac-1* after combined treatment with QTP and radiation (Fig. 6A and B). *In vitro*, surviving cells treated with radiation and QTP showed significantly increased migratory capacity (Fig. 6C and D), known to require remodeling of the cytoskeleton (22). The addition of atorvastatin inhibited *Rac-1* activation (Fig. 6A) and diminished the increased migratory capacity seen in cells surviving the combination treatment (Fig. 6C and D). Microtubules are part of the cytoskeleton, a structural network within the cell's cytoplasm which helps to support cell shape, as well as cell migration and cell division. Using a microtubule stain, we showed that the surviving cells treated with radiation and QTP had more filopodia (Fig. 6E, white arrowheads), which support cell migration by promoting cell–matrix adhesiveness at the leading edge, as well as increased numbers of tunneling nanotubes (TNT; Fig. 6E, yellow arrowheads**).** TNTs are known to mediate protein and mitochondrial transfer between distant cells as part of the radiation response of GBM cells and are suspected to drive GBM growth and treatment resistance (23, 24). This suggested more active *Rac-1*–mediated cytoskeleton remodeling in cells surviving the combination of radiation and QTP. Importantly, the observed effects on cytoskeleton remodeling were inhibited with the additional atorvastatin (Fig. 6E).

Finally, we performed loss-of-function experiments to demonstrate that radiation/QTP-induced *Rac-1* activation is indeed a driver for stemness maintenance in surviving GSCs. With the efficient transient knockdown of *Rac-1* in HK374 cells (Fig. 6F and G), we tested its role in both, clonogenicity survival and self-renewal capacity of the cells surviving radiation and/or QTP and the effects of atorvastatin addition. While treatment with radiation and QTP significantly reduced the formation of adherent clonal colonies (Fig. 6H and I), sphere-forming capacity (Fig. 6J) and stem cell frequency (Fig. 6K; Supplementary Tables S2 and S3), additional treatment with atorvastatin did not further diminish these three parameters after knockdown of *Rac-1*, thus suggesting that all three traits are *Rac-1*–driven in cells surviving radiation and QTP.

## Discussion

To date, surgery and radiotherapy remain the most effective treatment modalities against GBM. TMZ, the only chemotherapeutic agent added to the standard of care during the past two decades (25, 26), only marginally improves patient outcome and the median and long-term survival rates of patients suffering from GBM remain unacceptably low. Attempts to increase the tumoricidal efficacy of radiotherapy through, for example, alternative fractionation schemes or radiosensitizers have largely failed (27), in part due to the dispersion of glioma cells into the normal parenchyma beyond the visible tumor and because many compounds lacked BBB penetration or increased normal tissue toxicity. Likewise, targeted therapies had little effects as GBMs escape through utilization of alternate signaling pathways (28).

The DNA-damaging effects of radiation unfold in milliseconds after irradiation and the repair of DNA double-strand breaks is completed within the first few hours. Compounds that increase the amount of initial DNA damage or interfere with its repair mainly operate during this short timeframe. While it is desirable to eliminate all malignant cells this way, current therapies against GBM fail in achieving this goal and almost all patients succumb to progressive or recurrent disease. Over the past two decades, it became evident that a small population of CSCs, relatively resistant to radiotherapy and chemotherapy, maintains the growth of tumors (7, 29, 30) and shows enrichment after treatment (2, 31, 32). Recurrent or progressing gliomas are likely caused by GSCs that survive these sublethal treatments and the pathways that drive them to proliferate, invade, and repopulate the tumor are not necessarily identical with the pathways that helped them to survive the genotoxic insults.

With the discovery of radiation-induced phenotype conversion of non-stem cancer cells into CSCs, we have reported a novel major factor for radiation treatment failure (33). In an unbiased phenotypic high-throughput screen, we identified compounds that unlike TMZ, which induces cellular plasticity- prevented this unwanted effect of radiation, and we demonstrated that repurposing FDA-approved drugs identified in this screen prolonged median survival in PDOX models of glioma (3, 4).

The mevalonate pathway is one of the key metabolic pathways known to be dysregulated in GBM (34). Statins, developed to lower cholesterol levels in the periphery target *HMGCR*, the rate-limiting enzyme in this pathway and affect glioma biology in multiple ways, from interfering with the dependence of GBM on cholesterol to inhibition of farnesylation of Ras or geranylgeranylation of GTPases with effects on cell proliferation and survival, cell cycle progression, migration, and invasion (35). Recognized as a potential target, the mevalonate pathway has been probed in several preclinical and clinical studies repurposing statins against brain tumors with largely disappointing results. While statins showed robust antitumor activities *in vitro* (35–37), previous rodent pharmacokinetic studies were not able to achieve therapeutic drug concentrations in the central nervous system (CNS; refs. 38, 39). A recent clinical phase II study, which added atorvastatin to the current standard-of-care, radiotherapy and TMZ, showed no beneficial effects on progression-free or overall survival when compared with historic controls treated with radiotherapy and TMZ (40)

Clinically, the biodistribution of TMZ into the brain is less than 20% and peak concentrations after single doses of 75–200 mg/m^2^ were predicted to reach only 1.8–3.7 µg/mL or 9.2 to 19.1 µmol/L (41). At these concentrations, TMZ has only minimal if any effects *in vitro* and most preclinical studies use TMZ in the high micromolar to millimolar range (42, 43). Although TMZ is a known activator of the MAPK cascade, our *in vitro* data did not indicate effects of TMZ on the mevalonate pathway unless drug concentrations were raised to 1 mmol/L. The lack of upregulation of the mevalonate pathway in response to low TMZ concentrations agreed with the lack of clinical benefits of statins in combination with radiation and TMZ, which was not surprising given that 23% of the human body's cholesterol are located in the brain (44) and that GBM cells rely on exogenous cholesterol and not on cholesterol biosynthesis (17).

On the contrary, dopamine receptor antagonists activate the MAPK cascade via *GSK-3* at low concentrations and synergize with radiation by targeting tumor cell plasticity and glioma stem cells (3, 5). Designed as psychotropic drugs they easily cross the BBB and reach therapeutic concentrations in the CNS. Here we show that dopamine receptor antagonists robustly created a metabolic vulnerability in surviving cells through upregulation of the mevalonate pathway *in vivo*.

Tumors outpace cell loss with proliferation, thereby creating a need for macromolecular building blocks to not only replicate DNA but also to double the cellular mass before and during mitosis. To serve this need, cancer cells utilize aerobic glycolysis, known as the Warburg effect, to channel glucose into the biosynthesis of macromolecules including the mevalonate pathway to produce cholesterol. The cell cycle and the mevalonate pathway are tightly intervened with non-sterol isoprenoids initiating the transition from G_1_- to S-phase and cholesterol being required during the G_2_- and M-phase of the cell cycle. Very high cholesterol concentration is toxic for cells and cholesterol biosynthesis is limited by a negative feedback loop (45). And because GBM cells primarily rely on uptake of exogenous cholesterol (17), it is not surprising that the radiation/QTP-induced upregulation of the mevalonate pathway reported here only slightly increased cholesterol biosynthesis in GBM cells. Consequently, the inhibition of the cholesterol branch of the mevalonate pathway did not affect the self-renewal of surviving GBM cells beyond the effects of radiation and dopamine receptor antagonists. Instead of upregulating cholesterol production, surviving cells increased their migratory and invasive capacity through activation of *Rac-1*, a major regulator of cytoskeleton reorganization. A role for *Rac-1* in stemness and tumorigenicity had been previously reported for U87MG and U373 glioma cells (46) and our results confirmed these findings with a significant reduction in clonogenic survival, sphere-forming capacity and glioma stem cell frequency when *Rac-1* was knocked down. Likewise, the treatment of cells with a geranylation inhibitor or inhibition of *Rac-1* synergized with radiation and QTP in reducing the sphere-forming capacity of the cells.

We conclude that the upregulation of the mevalonate pathway is part of the immediate-early response to glioma treatments that converge in activating the MAPK cascade, effectively diminish the GSC pool and interfere with glioma cell plasticity. Surviving GSCs rely on activation of *Rac-1* through the mevalonate pathway to maintain stemness and to repopulate the tumors. This generates a metabolic vulnerability that can be exploited through the addition of statins to further improve the efficacy of radiotherapy. Furthermore, upregulation of the mevalonate pathway seemed to be restricted to tumor tissue, indicating the presence of a therapeutic window for statins in combination with radiation and repurposed, FDA-approved dopamine receptor antagonists. These effects on the mevalonate pathway are not seen after treatment with radiation and TMZ and could potentially explain why the addition of statins to the current standard of care has not translated into clinical benefit.

## Supplementary Material

Supplementary Data Supplemental Data
