# Supplementary material for: Activation of the Mevalonate Pathway in Response to Anti-cancer Treatments Drives Glioblastoma Recurrences Through Activation of Rac-1
Source: Cancer Res Commun. 2024 Jun 25;4(6):1566–80. doi: 10.1158/2767-9764.CRC-24-0049 (PMC11197925; doi:10.1158/2767-9764.CRC-24-0049)
Supplement: Supplementary Data — Supplemental Data [file crc-24-0049-s01.docx]

**Supplementary Materials for**

# Activation of the mevalonate pathway in response to anti-cancer treatments drives glioblastoma recurrences through activation of Rac-1

**Table of contents:**

- Supplementary Figure
- Supplementary Tables
- Supplementary Materials and Methods


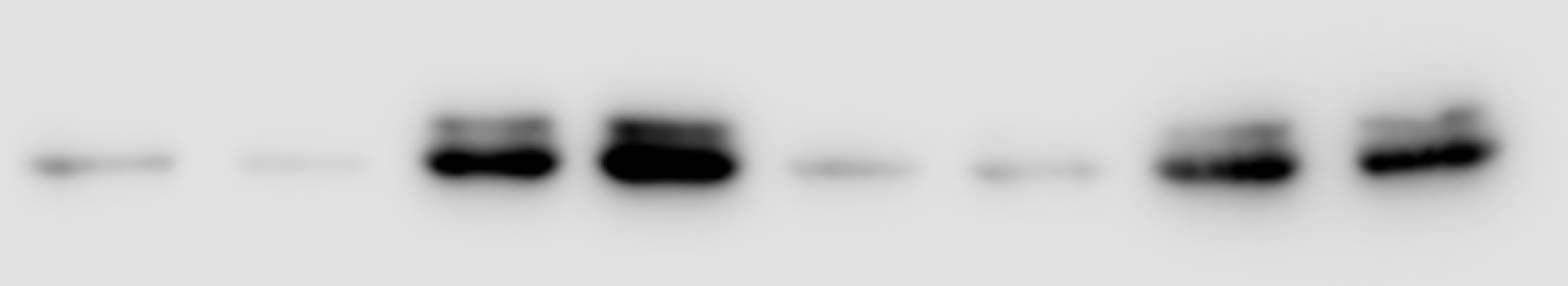

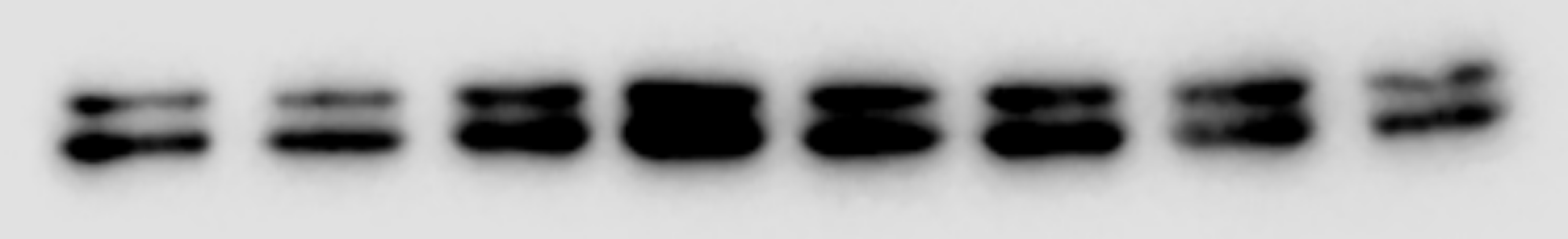


DMSO

30”

HK374

2h

4h

*p-ERK*

Total ERK

A

B


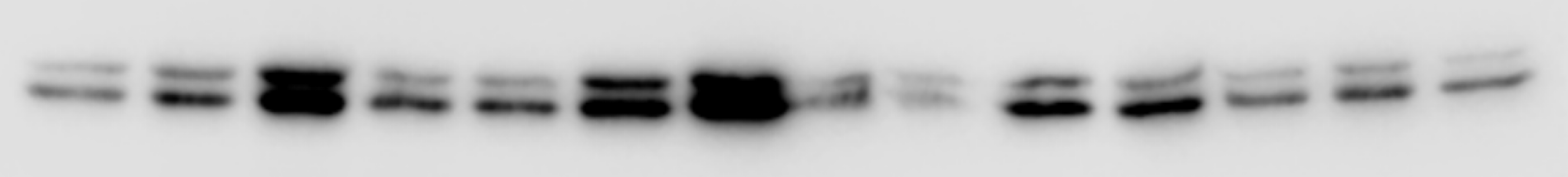

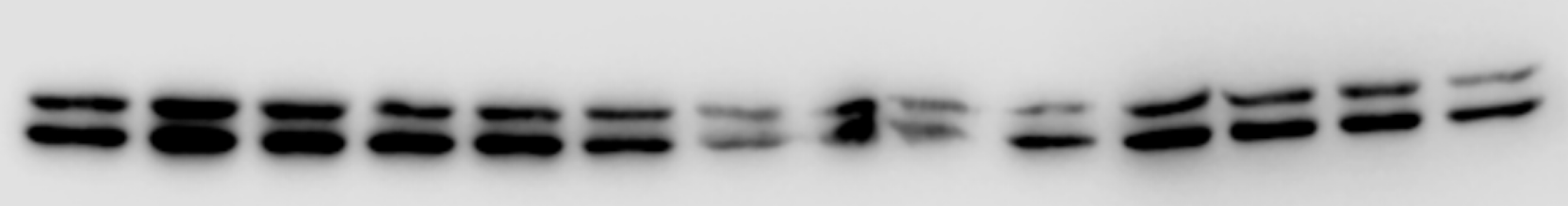


*p-ERK*

Total ERK

QTP

DMSO

30”

2h

4h

TMZ

30”

2h

4h

Vincristine

C

DMSO

*p-ERK*

30”

2h

4h

Vincristine

HK217

Total ERK


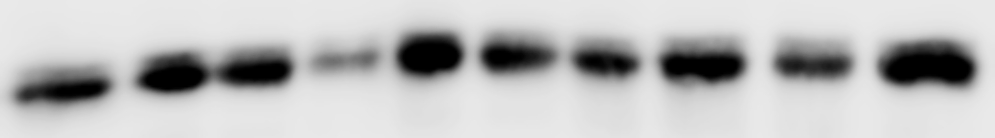

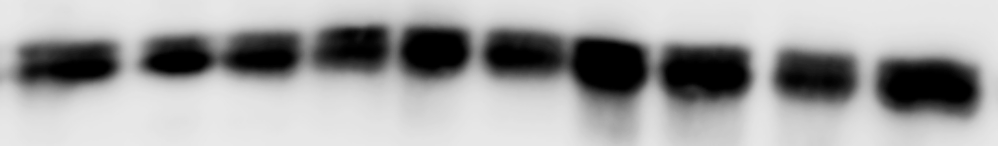


30”

2h

4h

QTP

30”

2h

4h

TMZ

Supplementary Figure 1.

**Supplementary Figure 1.** Western blotting of *p*-ERK and total ERK in HK374 cells **(A/B)** or HK217 cells **(C)** treated with QTP (10 µM), TMZ (1 mM) or Vincristine (250 nM) for 30 minutes, 2 hours, and 4 hours, with the solvent DMSO-treated cells as the control.

**Supplementary Table 1**

| Species | Gene name | Primer sequence (5’–3’) |
| --- | --- | --- |
| Human | HMGCR | Forward: TGATTGACCTTTCCAGAGCAAG  Reverse: CTAAAATTGCCATTCCACGAGC |
| Human | HMGCS1 | Forward: CATTAGACCGCTGCTATTCTGTC  Reverse: TTCAGCAACATCCGAGCTAGA |
| Human | HMGCS2 | Forward: TCCCTTTACCTCTCCACTCAC  Reverse: CCATAAGAGAAGGCACCAATCC |
| Human | DHCR7 | Forward: GCTGCAAAATCGCAACCCAA  Reverse: AGCTTTGGTTCTCTGCCTG |
| Human | DHCR24 | Forward: GCCGCTCTCGCTTATCTTCG  Reverse: GTCTTGCTACCCTGCTCCTT |
| Human | ACAT1 | Forward: CGGGCTAACTGATGTCTACAA  Reverse: CAAATTTCCCAGCTTCCCATG |
| Human | ACAT2 | Forward: CCCAGAACAGGACAGAGAATG  Reverse: AGCTTGGACATGGCTTCTATG |
| Human | SREBF2 | Forward: AACGGTCATTCACCCAGGTC  Reverse: GGCTGAAGAATAGGAGTTGCC |
| Human | INSIG1 | Forward: CCTGGCATCATCGCCTGTT  Reverse: AGAGTGACATTCCTCTGGATCTG |
| Human | SQLE | Forward: GATGATGCAGCTATTTTCGAGGC  Reverse: CCTGAGCAAGGATATTCACGACA |
| Human | MVD | Forward: CGTGGCATCGGTGAACAACT  Reverse: GTGTAGGCTAGGCAGGCATA |
| Human | MSMO1 | Forward: TATGCTGGTTCTCGGCATCAT  Reverse: CCAAAAATTCGATCCCACCATGT |
| Human | SC5D | Forward: CATACGTGTATCCAGCCAC  Reverse: AAGAACAGTGCAACAGTAAGA |
| Human | FDFT1 | Forward: CCACCCCGAAGAGTTCTACAA  Reverse: TGCGACTGGTCTGATTGAGATA |
| Human | FDPS | Forward: TGTGACCGGCAAAATTGGC  Reverse: GCCCGTTGCAGACACTGAA |
| Human | JunB | Forward: GGACACGCCTTCTGAACG  Reverse: CGGAGTCCAGTGTGGTTTG |
| Human | JunD | Forward: CCTCAGCCACGTCAACAG  Reverse: CACCCTCTCCAAGTCCG |
| Human | FosB | Forward: AGCTAAATGCAGGAACCGG  Reverse: ACCAGCACAAACTCCAGAC |
| Human | Rac1 | Forward: GGTGAATCTGGGCTTATGGG  Reverse: TCAGGATACCACTTTGCACG |
| Human | PPIA | Forward: ATGCTGGACCCAACACAAAT  Reverse: TCTTTCACTTTGCCAAACACC |
| Mouse | HMGCR | Forward: GCCCTCAGTTCAAATTCACAG  Reverse: TTCCACAAGAGCGTCAAGAG |
| Mouse | HMGCS1 | Forward: TGTTCTCTTACGGTTCTGGC  Reverse: AAGTTCTCGAGTCAAGCCTTG |
| Mouse | HMGCS2 | Forward: GTACCTTGAACGAGTGGATGAG  Reverse: GGTGGGATTTTAAGCAGATGC |
| Mouse | DHCR7 | Forward: TTATTCCTGGCTTCCTGACTTC  Reverse: CAGAGGATGTGGGTAATGAGC |
| Mouse | DHCR24 | Forward: AGAACTACCTGAAGACAAACCG  Reverse: GAAGAGGTAGCGGAAGATGG |
| Mouse | ACAT1 | Forward: AGCACACTGAACGATGGAG  Reverse: CGCAAGTGGAAAATCAATGGG |
| Mouse | ACAT2 | Forward: CTGGAGGCATGGAGAATATGAG  Reverse: CATGTGGTAGTTGTGAAAGGC |
| Mouse | SREBF2 | Forward: CCCTATTCCATTGACTCTGAGC  Reverse: CACATAAGAGGATTCGAGAGCG |
| Mouse | INSIG1 | Forward: GATTACCATCGCCTTCCTAGC  Reverse: CGTCCTATGTTTCCCACTGTG |
| Mouse | SQLE | Forward: CCCCAAAACACAAAATCCTCAG  Reverse: GCAATGCCAAGAAAAGTCCAC |
| Mouse | MVD | Forward: GCTCCGAATCCTTATCCTTGTG  Reverse: GGGTCATCTCCTTCATGCG |
| Mouse | MSMO1 | Forward: ATTTCCTGCACAGACTCCTTC  Reverse: AGAATCAGGGTTTCCAAGGG |
| Mouse | SC5D | Forward: CCGTCTCACTGTTCCTGC  Reverse: GCCCCTATGAATCCAGTAGATC |
| Mouse | FDFT1 | Forward: GTGTGGGATGGCAGAATTTG  Reverse: GGCAGAGAATAGACGAGAAAGG |
| Mouse | FDPS | Forward: TCTTTCTACCTGCCTATTGCG  Reverse: CTCCAAAGAGATCAAGGTAGTCG |
| Mouse | JunB | Forward: GGACACGCCTTCTGAGAG  Reverse: GAGTCCAGTGTGTGAGCTG |
| Mouse | JunD | Forward: AACAGAAAGTCCTCAGCCAC  Reverse: AGTCTCGAAAGAGTCCGGG |
| Mouse | FosB | Forward: AGTCTCAGTACCTGTCTTCGG  Reverse: CACGAGCCACTGAAGATCC |
| Mouse | GAPDH | Forward: AGGTCGGTGTGAACGGATTTG  Reverse: CCCTGGCACATGAATCCTGG |

**Supplementary Table 2. Confidence intervals for stem cell frequency (%).**

| Groups | Lower | Estimate | Upper |
| --- | --- | --- | --- |
| siCtrl_DMSO | 7.246377 | 9.90099 | 13.45895 |
| siCtrl_RT + DMSO | 3.584229 | 4.901961 | 6.697924 |
| siCtrl_RT + QTP | 0.785546 | 1.079914 | 1.483239 |
| siCtrl_RT + QTP + Atorvastatin | 0.403877 | 0.555247 | 0.762893 |
| siRac-1_DMSO | 4.608295 | 6.329114 | 8.613264 |
| siRac-1_RT + DMSO | 2.083333 | 2.857143 | 3.913894 |
| siRac-1_RT + QTP | 1.48368 | 2.040816 | 2.800336 |
| siRac-1_RT + QTP + Atorvastatin | 0.816327 | 1.122334 | 1.541545 |

**Supplementary Table 3. Pairwise tests for differences in stem cell frequencies.**

| Group 1 | Group 2 | Chisq | DF | Pr(>Chisq) |
| --- | --- | --- | --- | --- |
| siCtrl_DMSO | siCtrl_RT + DMSO | 8.81 | 1 | 0.003 |
| siCtrl_RT + DMSO | siCtrl_RT + QTP | 41 | 1 | 1.51e-10 |
| siCtrl_RT + QTP | siCtrl_RT + QTP + Atorvastatin | 7.86 | 1 | 0.00507 |
| siCtrl_DMSO | siRac-1_DMSO | 3.64 | 1 | 0.0562 |
| siCtrl_RT + DMSO | siRac-1_RT + DMSO | 4.95 | 1 | 0.0261 |
| siRac-1_ DMSO | siRac-1_RT + DMSO | 10.7 | 1 | 0.00105 |
| siRac-1_RT + DMSO | siRac-1_RT + QTP | 1.92 | 1 | 0.165 |
| siRac-1_RT + QTP | siRac-1_RT + QTP + Atorvastatin | 6.06 | 1 | 0.0138 |

**Supplementary Materials and Methods**

*Ripa lysis buffer*

10 mM Tris-HCl (pH 8.0), 1 mM EDTA, 1 % Triton X- 100, 0.1 % Sodium Deoxycholate, 0.1 % SDS, 140 mM NaCl, 1 mM PMSF) containing protease inhibitor (#A32955, Thermo Fisher Scientific, Waltham, MA) and phosphatase inhibitor (#A32957, Thermo Fisher Scientific).

*Western blot 1X Stacking buffer*

1 M Tris-HCl, 0.1% SDS, pH 6.8, 1X Separating buffer - 1.5 M Tris-HCl, 0.4% SDS, pH 8.8

*Western blot 1X Running buffer*

12.5 mM Tris-base, 100 mM Glycine, 0.05% SDS

*TBST*

20 mM Tris-base, 150 mM NaCl, 0.2% Tween-20, 5% bovine serum albumin (BSA)

*Western Blotting*

HK374 and HK217 cells were serum starved overnight and the following day treated with 10 μM QTP, 1 mM TMZ, or 250 nM Vincristine for one hour and then irradiated with a single dose of 10 Gy. Two hours after irradiation, the cells were lysed in 150 μl of ice-cold RIPA lysis buffer. The protein concentration in each sample was determined by BCA protein assay and samples were denaturated in 4X Laemmli sample buffer (Bio-Rad) containing 10% β- mercaptoethanol for 10 mins at 95°C. Equal amounts of protein were loaded onto 10% SDS-PAGE gels, initially at 50 V for 30 min followed by 100 V for 2 hours. Samples were then transferred onto 0.45 μm nitrocellulose membrane (Bio-Rad, Hercules, CA) for 2 hours at 80 V. Membranes were blocked in 1X TBST for 30 min and then incubated with primary antibodies in 1X TBST containing 5% BSA overnight at 4°C with gentle rocking. Membranes were then washed three times for 5 min each with 1X TBST and incubated with secondary antibodies, 1:2000 anti-mouse or anti-rabbit horseradish peroxidase (HRP; Cell Signaling) in TBST for 2 hours at room temperature with gentle rocking. Membranes were washed again three times for 5 min each with 1X TBST. Pierce ECL Plus Western Blotting Substrate (Thermo Fisher Scientific) was added to each membrane and incubated at room temperature for 5 min. The blots were then scanned with Odyssey Fc Imaging system (LI-COR, Lincoln, NB). β-actin was used as a loading control. The ratio of the gene of interest over its endogenous control was calculated and expressed as relative intensity.

*Quantitative Reverse Transcription-PCR*

Total RNA was isolated using TRIZOL. cDNA synthesis was carried out using the SuperScript Reverse Transcriptase IV (Invitrogen). Quantitative PCR was performed in the QuantStudio^TM^ 3 Real-Time PCR System (Applied Biosystems, Carlsbad, CA, USA) using the PowerUp^TM^ SYBR^TM^ Green Master Mix (Applied Biosystems). *C*_t_ for each gene was determined after normalization to PPIA and ΔΔ*C*_t_ was calculated relative to the designated reference sample. Gene expression values were then set equal to 2^−ΔΔCt^ as described by the manufacturer of the kit (Applied Biosystems). All PCR primers were synthesized by Invitrogen with PPIA as the housekeeping gene.

*Mass spectrometry*

Atorvastatin or Simvastatin Detection: Chromatographic separations were performed on a 100 x 2.1 mm Phenomenex Kinetex C18 column (Phenomenex, Torrence, CA) using the 1290 Infinity LC system (Agilent, Santa Clara, CA). The mobile phase was composed of solvent A: 0.1% formic acid in Milli-Q water, and B: 0.1% formic acid in acetonitrile. Analytes were eluted with a gradient of 5% B (0-4 min), 5-99% B (4-32 min), 99% B (32-36 min), and then returned to 5% B for 12 min to re-equilibrate between injections. Injections of 20 µL into the chromatographic system were used with a solvent flow rate of 0.10 mL/min.

            Mass spectrometry was performed on a 6460 triple quadrupole LC/MS system (Agilent). Ionization was achieved by using positive ion electrospray ionization and data acquisition was made in multiple reaction monitoring mode. Atorvastatin was monitored with the transition from m/*z*559.2→250 with fragmentor settings at 45 V and a collision energy of 17 and an accelerator voltage of 4V, and for Simvastatin m/*z* 419.2→198.2 with fragmentor settings at 85 V and a collision energy of 5 and an accelerator voltage of 4 V.

*Irradiation*

Cells were irradiated at room temperature using an experimental X-ray irradiator (Gulmay Medical Inc. Atlanta, GA) at a dose rate of 5.519 Gy/min. Control samples were sham-irradiated. The X-ray beam was operated at 300 kV and hardened using a 4 mm Be, a 3 mm Al, and a 1.5 mm Cu filter and calibrated using NIST-traceable dosimetry. Corresponding controls were sham irradiated.

For *in vivo* irradiation experiments, mice were anesthetized prior to irradiation with an intra-peritoneal injection of 30 µL of a ketamine (100 mg/mL) and xylazine (20 mg/mL) mixture (4:1) and placed on their sides into an irradiation jig that allows for irradiation of the midbrain while shielding the esophagus, eyes, and the rest of the body. For survival experiments, animals implanted with GL261 cells received a single dose of 10 Gy on day 7 or 5 fractions of 3 Gy each starting 7 days after tumor implantation. Animals injected with HK374 glioma specimen received a single dose of 10 Gy on day 3 after tumor implantation.

*Antibodies*

*p*-ERK (#4370S, 1:1000, Cell Signaling, Danvers, MA)

*p*-P38 (#4511S, 1:1000, Cell Signaling)

t-ERK (#5013S, 1:1000, Cell Signaling)

t-P38 (#8690S, 1:1000, Cell Signaling)

Rac1 (#ARC03, 1:500, Cytoskeleton, Denver, CO )

β-actin (#3700S, 1:1000, Cell Signaling)

*Mevalonate pathway inhibitors*

Atorvastatin (#10493, Cayman Chemical, Ann Arbor, MI)

Simvastatin (#S1792, Selleck Chemicals, Houston, TX)

zaragozic acid (squalene synthase inhibitor; #17452, Cayman Chemical,)

GGTI-298 (GGTase inhibitor; #16176, Cayman Chemical)

YM-53601 (squalene synthase inhibitor; #18113, Cayman Chemical)

Lonafarnib (farnesyltransferase inhibitor, #SML1457, Sigma, St. Louis, MO)

Rhosin (RhoA-specific inhibitor; #555460, Sigma),

Y27632 (ROCK1/2 inhibitor; #S1049, Selleck Chemicals)

EHop-016 (Rac GTPase inhibitor; #S7319, Selleck Chemicals)

CID44216842 (Cdc42-selective inhibitor; #S6000, Selleck Chemicals)

*Durgs and Chemicals*

quetiapine (#KS-1099, Key Organics, Cornwall, UK)

Temozolomide ( #14163, Cayman Chemical)

Vincristine (#HY-N0488, MedChem Express, Monmouth Junction, NJ)

Ketamine (Phoenix, MO)

Xylazine (AnaSed, Lake Forest, IL)

TRIZOL Reagent (Invitrogen, Waltham, MA)

Lipofectamine duplex (#13778075, Thermo Fisher Scientific)

*Kits and other Materials*

Cholesterol/Cholesterol Ester-GLo^TM^ assay (#J3190, Promega, Madison, WI)

Free Fatty Acid assay (ab65341, Abcam, Cambridge, UK)

BCA protein assay (Thermo Fisher Scientific)

ViaFluor® 488 live cell microtubule stains (#70062, Biotium, Fremont, CA)

**Poly-D-Lysine coated dishes (No. 1.5 Coverslip, 10 mm Glass Diameter, Poly-D-Lysine coated;** #P35GC-1.5-10-C, MatTek **Ashland, MA)**

12-well Transwell^®^ insert with 8 µm pore size (Corning, New York, NY)
